# Bicomponent Carbon Fibre within Woven Fabric for Protective Clothing

**DOI:** 10.3390/polym12122824

**Published:** 2020-11-27

**Authors:** Stana Kovačević, Snježana Brnada, Ivana Schwarz, Ana Kiš

**Affiliations:** 1 Department of Textile Design and Menagement, Faculty of Textile Technology, University of Zagreb, Prilaz Baruna Filipovića 28a, 10000 Zagreb, Croatia; stana.kovacevic@ttf.unizg.hr (S.K.); snjezana.brnada@ttf.unizg.hr (S.B.); 2 Textile Company Čateks d.d., 40000 Čakovec, Croatia; a.kis@cateks.hr

**Keywords:** protective clothing fabric, bicomponent carbon fibre, electrostatic resistance, biaxial cyclic stresses, physical-mechanical properties

## Abstract

For the purpose of this research, six types of woven fabrics with different proportions of bicomponent carbon fibres (CF), differently distributed in the fabric, were woven and tested. Fibre composition in the core and sheath was determined with X-ray spectroscopy (EDS). Two types of bicomponent CF were selected which are characterised by different proportions of carbon and other polymers in the fibre core and sheath and different cross-sections of the fibres formed during chemical spinning. Physical-mechanical properties were investigated, as well as deformations of fabrics after 10,000, 20,000 and 30,000 cycles under biaxial cyclic stress on a patented device. Tests of the surface and vertical electrostatic resistance from fabric front to back side and from the back side to the fabric front were conducted. According to the obtained results and statistical analyses, it was concluded that the proportion of CF affects the fabric’s physical and mechanical properties, the electrostatic resistance as well as the deformations caused by biaxial cyclic stresses. A higher proportion of CF in the fabric and a higher proportion of carbon on the fibre surface, gave lower electrostatic resistance, i.e., better conductivity, especially when CFs are woven in the warp and weft direction. The higher presence of CF on the front of the fabric, as a consequence of the weave, resulted in a lower surface electrostatic resistance.

## 1. Introduction

Carbon fibre can today be found in a wide range of technical textile applications like medical applications and filtration, aerospace, wind energy, as well as the automotive industry [1]. Thanks to their properties, carbon fibres are frequently used for production of protective textiles i.e., woven fabric for protective clothing.

Fabrics for protective clothing vary greatly and depend on the purpose or the type of protection they must provide when exposed to extreme conditions. In order to develop woven fabrics that will be multipurpose for simultaneous protection, e.g., electrostatic charge, it is necessary to intensively investigate all the properties from fibres and yarns to finished woven fabric. Good insulating properties of polymeric materials, i.e., very high surface electrical resistance and resistance to current flow, result in the accumulation of electrostatic charge. Namely, by rubbing a polymer and a neutral surface or by rubbing two polymers and after separating the surfaces, one of the components becomes positively charged and the other negatively charged. The human body has low enough resistance to act as a conductor and if isolated from the ground, can accumulate an electrostatic charge. The charge can be produced by walking over an insulated floor or by contact with charged devices or materials. It can also be caused by induction due to charged clothing or nearby objects. The electrostatic charge results in a risk of sparking. Control of unwanted static electricity is necessary in areas of flammable or explosive atmospheres. In such cases, the person must be earthed directly or via conductive footwear i.e., via a conductive floor. In areas with sensitive materials or flammable mixtures, it is mandatory to wear protective clothing that has appropriate electrostatic properties. Since the dielectric constant of hydrophilic polymers is highly dependent on moisture, due to the influence of high water permittivity, they become antistatic in wet conditions and static in dry conditions [2,3].

Protecting the body from electrostatic charge is extremely important when a person finds themself in low humidity conditions and in hydrophilic-fibre clothing. Materials, which easily exchange electrons (or charges) between atoms, are called conductors because they possess freely moving electrons. Examples of conductors are metals, carbon and a layer of sweat on the human body. Materials that do not exchange electrons are called insulators, including most textile raw materials, especially those that contain a lower percentage of moisture.

The threat of electrostatic charge is not visible and can be compared to infection of the human body by viruses or bacteria. Although they are invisible to unaided eye, they can cause great damage even before their presence is noticed. Therefore, preventive “vaccination” in the form of electrically conductive protective clothing is required. Textile fibres mostly belong to a group of insulators, especially in a dry environment, so implementation of additional fibres that have conductivity properties are needed. Since these are protective clothing, comfort is also important when worn for several hours and during activities when different and multiple cyclic stresses occur.

In order to develop a unique fabric that will provide protection according to strictly set norms, which will be strong and resistant enough and at the same time breathable and comfortable for several hours of wearing, it is necessary to define the starting point of the research. It is in this case the development of new and at the same time simple woven fabric structures, using appropriate textile raw materials. The first task is to select the appropriate fibres that will, by their properties, create protection from predefined extreme conditions [4,5,6].

In special areas where very sensitive materials or flammable mixtures are handled (e.g., on platforms), it is very important that staff wear clothing that has appropriate electrostatic properties. Static electricity on textiles usually has a negative effect that can cause health-hazardous situations, and problems can primarily be associated with the occurrence of electrical induction. In contact with a garment charged with static electricity, the human body acts as a conductor, so a charge is induced in it, which can be felt in contact with a metal object. In such a case, an electrical discharge occurs which feels like a burning sensation and an electric spark may also occur. To prevent the negative effects of static charging, protective clothing fabrics have requirements for adequate electrical conductivity [7,8,9].

Except to protect the body from the dangers of static electricity and provide wearing comfort, protective clothing must also provide resistance to multidirectional stress and a certain durability. Therefore, woven fabrics intended for protective clothing are mostly made of fibres that will give satisfactory properties, most often in combination with other fibres, as a mixture of natural and synthetic fibres. Natural fibres will provide comfort and breathability, while synthetic fibres provide resistance and longevity (carbon fibre (CF) antistatic conductivity). Therefore, it is good to combine different raw materials for protective clothing that will meet all requirements even after longer wearing and maintenance [10,11,12].

Bicomponent CFs act by electrostatically neutralizing the woven fabric surface by inducing a portion of the charge from the fabric surface into a conductive, carbon core. The conductive carbon core attracts an electric field from the fabric and neutralises the free charge on the surface. In doing so, a fabric containing a bicomponent CF does not necessarily have to be completely grounded to neutralise the surface. Fabrics with lower electrostatic conductivity, or higher vertical resistance, provide a property that stops the electrostatic conductivity towards the body. For special purposes, such as protective clothing for welders (with a voltage below 100 V), a high vertical resistance may be required (e.g., greater than 105 Ω), which will allow a certain degree of isolation of the body from the electrostatic charge.

Electrical resistance is a physical property that expresses the ratio of voltage and strength of electric current, which is a constant value for many materials. Due to their filamentous shape and fineness, many fibres and textile materials made from them, are often charged with static electricity during use. Textile fibres have characteristic electrical properties: if they are completely dry they act as good insulators, i.e., they have high electrical resistance, while in the wet state they have good conductivity. When it comes to the electrical conductivity requirements of clothing/woven fabrics, it is necessary to distinguish electrostatic surface and vertical resistance [3,13,14].

The aim of this research is to investigate the influence of the proportion of biocomponent CFs on the efficiency of static electricity conductivity. By using different polymers in the bicomponent fibre and their different positions in the core and sheath, as well as different cross-sectional shapes, the influence on the surface conductivity efficiency and on the physical-mechanical properties of woven fabrics will be investigated. The research was conducted in order to find the most acceptable fabric that will be characterised by exceptional properties of electrostatic conductivity, but also exceptional physical and mechanical characteristics.

## 2. Methods

**Figure 1:**
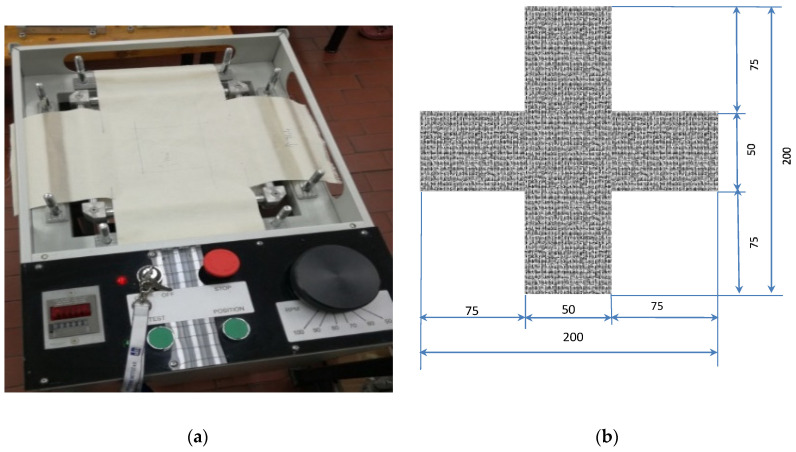
(**a**) Innovative cyclic biaxial stress measuring device, patent: HR P20150735; (**b**) sample prepared for cyclic stresses with dimensions in mm.

Six fabric samples intended for protective clothing for protection against static electricity were woven. When designing woven fabrics, the proportion of CFs in the yarn and their share in the fabric changed. Fabric samples differ by the proportion and position of CF in the fabric. All fabric samples were woven from yarns of the same raw material, fineness and density. The weave of the fabrics was twill 2/2 (samples 1–4) and twill 1/3 (samples 5 and 6), while the thickness and weight of the fabric varied according to the samples. CFs are woven as a multifilament yarn of fineness 30 tex in the weft direction (samples 1–3 and 5), in the warp and weft direction (sample 4) and as cut fibres twisted in the mixture in the warp and weft direction (sample 6). A more detailed presentation can be found in Tables 1 and 2. The tests were performed chronologically according to standardised methods and on the listed devices, as follows:

1. Cyclic biaxial stress (10,000, 20,000 and 30,000 cycles) on an innovative device (patent: HR P20150735) (innovative device, consensual patent No. PK20150735, with the State Intellectual Property Office of the Republic of Croatia, Zagreb, Croatia), details shown in Figure 1.

The test conditions were: preload of the samples in both directions—150 N, number of stress cycles—70/min. On this device, stressing can be uniaxial and biaxial, so the sample was fixed with two (opposite) or four clamps, with the possibility of adjusting the preload of the sample. Biaxial cyclic stress was used for these tests. Cyclic stress was achieved by a metal plate (200 × 200 mm) with side rollers, which acts with an applied force from below on a horizontally placed sample. The plate with the side rollers moves in the up–down direction and at the moment of moving upwards it stresses the sample and by returning the plate to the lower (initial) position, the sample is released from the stress. By cyclic movements of the plate, cyclic, biaxial stress of the sample is performed, always for the same deflection, which can be regulated. The force acting on the sample does not change during the sample elongation, which allows constant stress, regardless of the occurrence of deformations expressed by changing the sample dimensions. The maximum upper and lower position of the plate with side rollers changes during the test and depends on the elongation of the material, i.e., on the deformation occurrence. If the material elongation is greater the plate will be raised higher, so its maximum points in the upper and lower position will be higher, and vice versa. Achieving the displacement of the plate to a certain height is made possible by a spring which always maintains the same preload of the plate in the lower position. The device has the ability of speed regulation (up to 100 cycles/min) or the number of cycles per unit time, which allows more intensive research on material fatigue.

**Figure 2:**
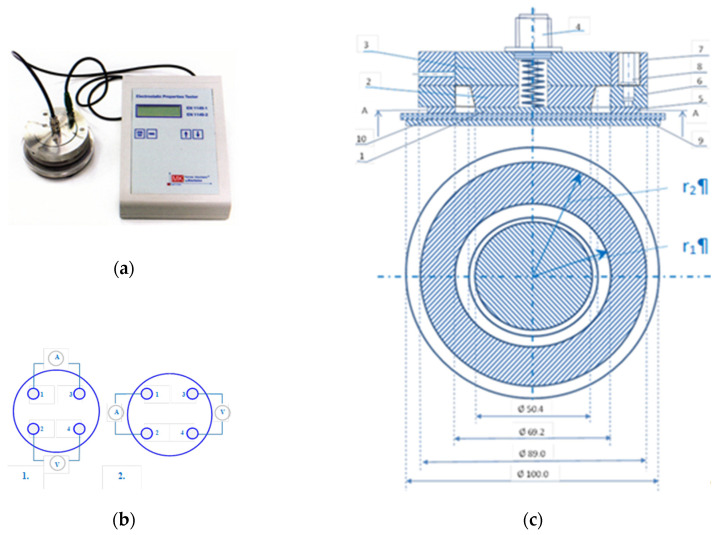
(**a**) Electrostatic resistance measuring device; (**b**) display of measuring electrodes for measuring surface (1) and vertical resistance (2); (**c**) scheme of measuring part: 1—test electrode, 2—insulating disk, 3—conductive plate, 4—coaxial socket for conduction, 5—circular electrode, 6—insulating ring, 7—ring, 8—connector, 9—base plate, 10—sample.

2. Electrostatic conductivity, i.e., fabric surface resistance, EN 1149-1, and electrostatic resistance through fabric or vertical resistance, EN 1149-2, on an electrostatic resistance tester, Mesdan, Puegnago del Garda, Italy, model STATIC LAB 291B (Figure 2) [15]. Between the two electrodes, the voltage drop caused by the surface resistance (between electrodes 1–3 and 2–4) (Figure 2b, 1) and vertical resistance (between electrodes 1–2 and 3–4) (Figure 2b, 2) is measured in the sample. Based on the obtained test results, the average surface electrostatic resistance (resistance *Rh*_(1)_ and *Rh*_(2)_, repetitive current I1-3, I2-4 and voltage U1-3, U2-4) and vertical electrostatic resistance (resistance *Rv*_(1)_ and *Rv*_(2)_, repetitive current I1-2, I3-4 and voltage U1-2 and U3-4) were obtained [12].

The surface resistance measured on the front (1) and on the back side (2) of the sample is expressed by the equation:

(1) $$Rh_{\left(1\right)}=\frac{U_{1-3}}{I_{2-4}}\;and\;Rh_{\left(2\right)}=\frac{U_{2-4}}{I_{1-3}},$$

A vertical resistance:

(2) $$Rv_{\left(1\right)}=\frac{U_{1-3}}{I_{2-4}}\;and\;Rv_{\left(2\right)}=\frac{U_{2-4}}{I_{1-3}},$$

The calculated resistance *ρ* (Ω) is obtained according to the following equation:

(3) ρ=k×R,

*R*—measured resistance (Ω)

*K*—geometric factor that depends on the radius of the electrode and can be obtained by the equation:

(4) $$k=\frac{2\pi }{log_{e}\left(\frac{r_{2}}{r_{1}}\right)},$$

for the used electrodes *k* = 19.8

*r*_1_—radius of the inner electrode (mm)

*r*_2_—inner radius of the outer electrode (mm)

**Figure 3:**
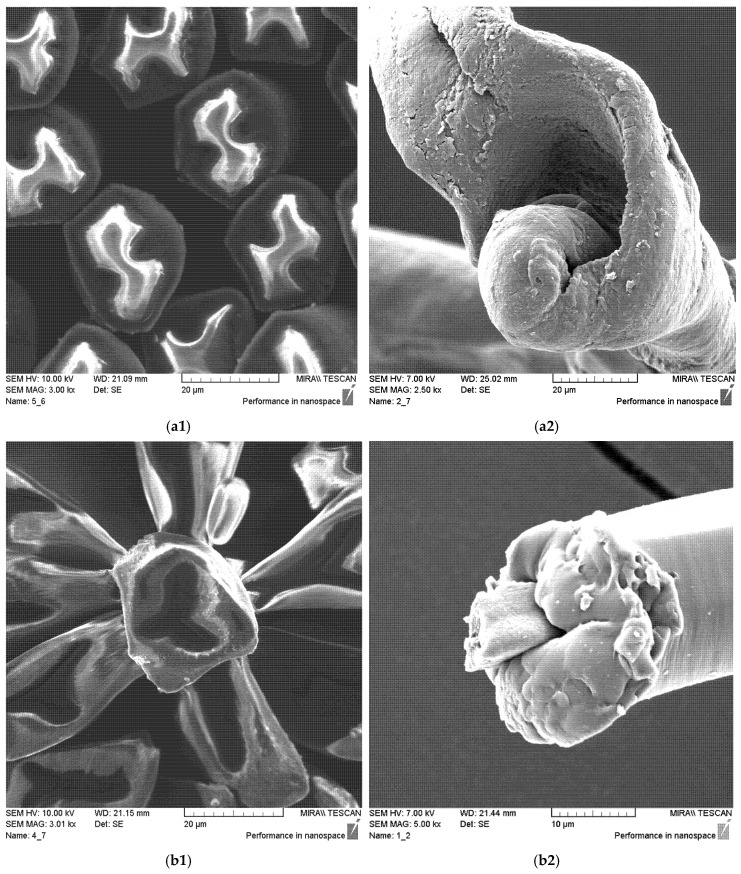
Bicomponent carbon fibre (CF) represented in: samples 1–4 (magnification: (**a1**) ×3000, (**a2**) ×5000); samples 5–6 (magnification: (**b1**) ×3000, (**b2**) ×5000).

**Figure 4:**
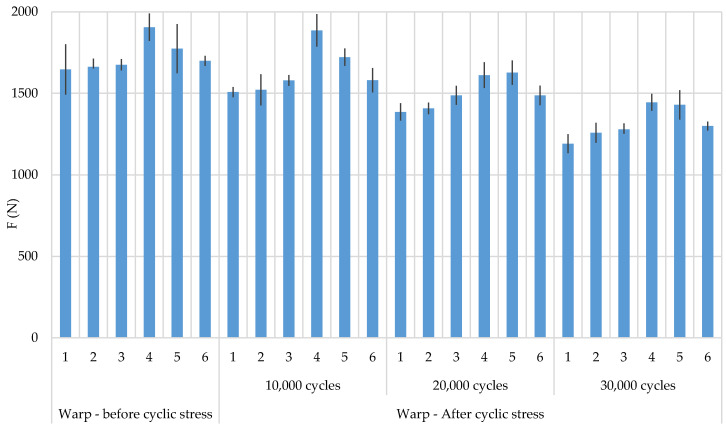
Breaking force of samples in warp direction, before and after subjection to cyclic loads, with error display.

**Figure 5:**
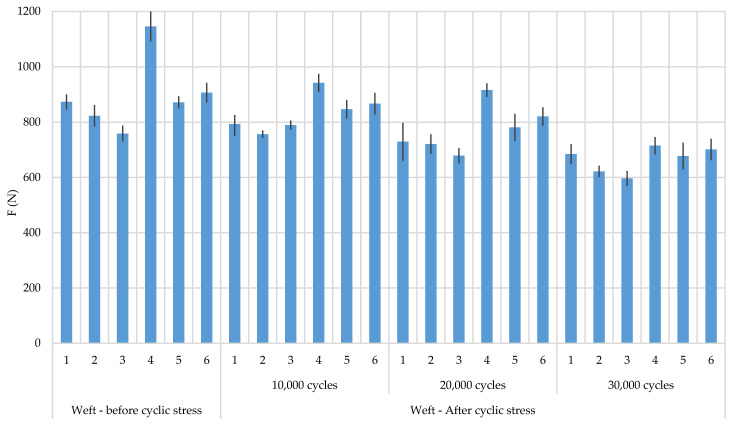
Breaking force of samples in weft direction, before and after subjection to cyclic loads, with error display.

3. A cross-sectional view of CF, on a scanning electron microscope (SEM) tt. Tescan, MIRA/LMU (Tescan Orsay Holding, a.s., Brno, Czech Republic) (Figure 3).

4. Position of CFs spun into yarn and fabric (Table 2).

5. Breaking forces and elongation at break of woven fabric, ISO 13934-1:2013, on a Textechno dynamometer, model Statimat M (Textechno H. Stein GmbH & Co. KG, Moenchengladbach, Germany) before and after cyclic load on prepared samples according to EN ISO 2062 (Tables 3 and 4, Figures 4 and 5).

6. Statistical analyses and comparisons of individual results were performed and are shown in Figures 8–10.

## 3. Results and Discussion

**Figure 6:**
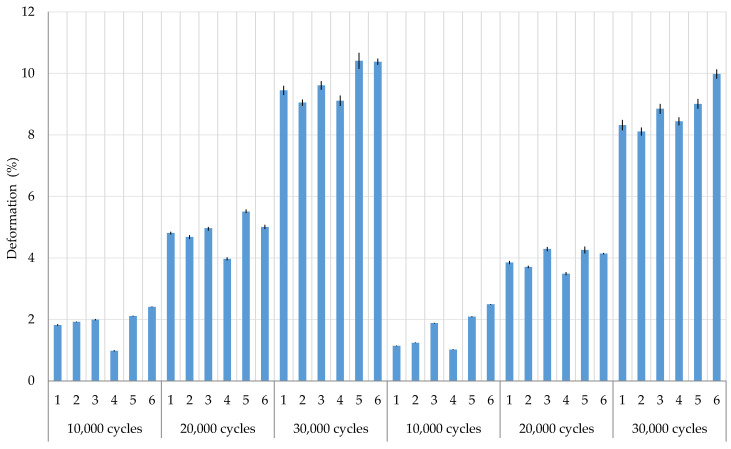
Woven fabric deformations according to the number of cycles with error display.

**Figure 7:**
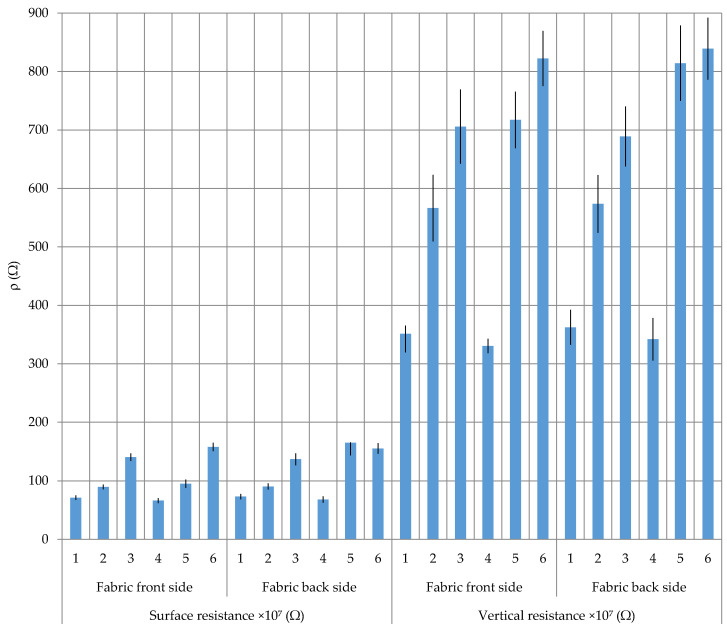
Surface and vertical resistance of the woven fabrics from the front to the back side, with error display.

**Figure 8:**
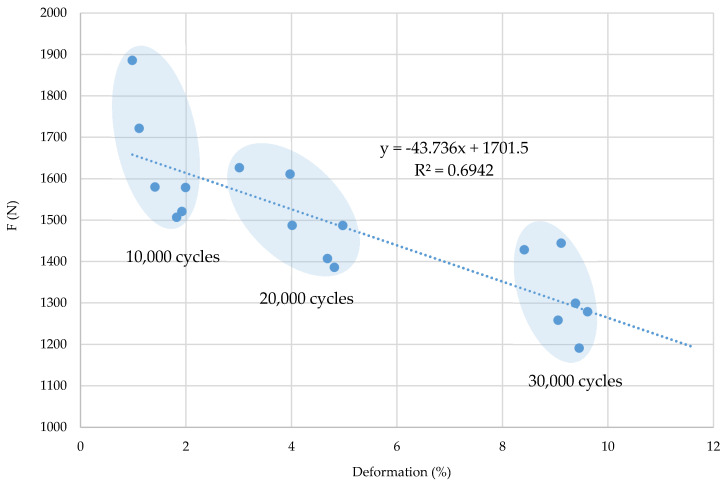
Correlation coefficient (of breaking forces and deformations), equation and regression line of samples in the warp direction.

**Figure 9:**
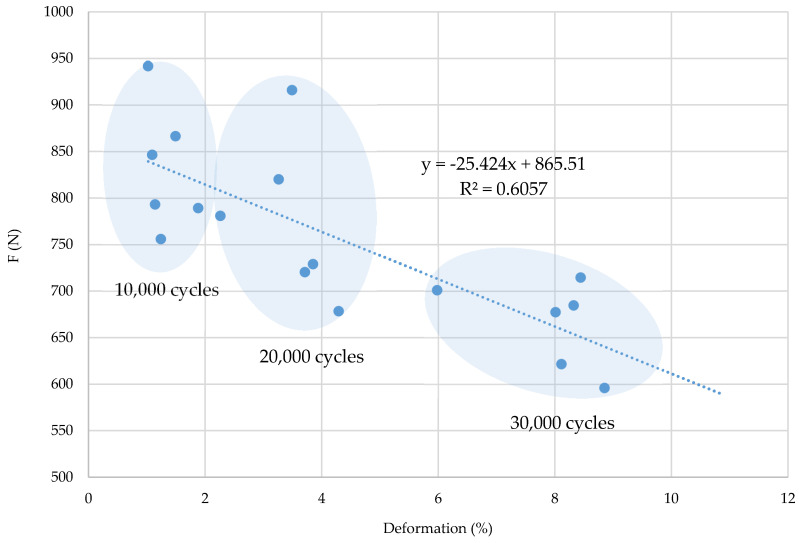
Correlation coefficient (of breaking forces and deformations), equation and regression line of samples in the weft direction.

**Figure 10:**
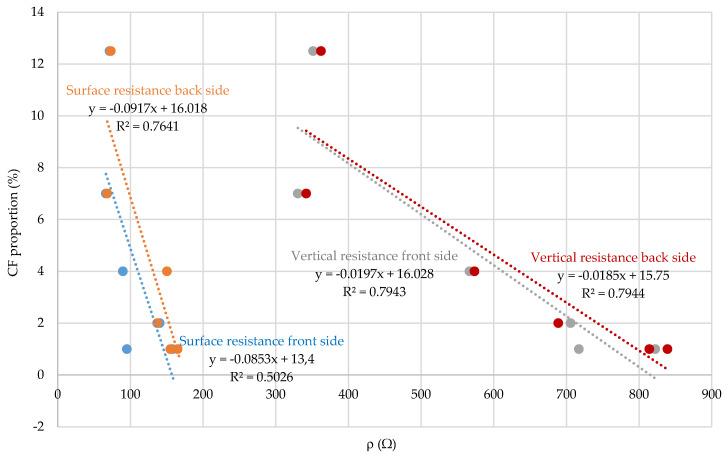
Correlation coefficient, equation and regression line of electrostatic resistance and CF proportion in the woven fabric.

In this paper, six samples of woven fabrics composed of polyester and cotton or modacrylic and cotton were made. All fabric samples contain a smaller proportion of bicomponent CFs and differ in the method of spinning, in yarns and in positions in the woven fabric. All test results are shown in Tables 1–6 and Figures 3–10.

### 3.1. Bicomponent CF: Cross-Sections, Proportion and Position of Fibres in the Fabric

In order for CF to be able to meet the specifications for wearing protective clothing and to fulfil its task, which is electrostatic conductivity, a certain “upgrade” is needed, which is achieved by spinning in the form of bicomponent fibre. By combining carbon with a polymer that can be arranged in the cross-section of the fibre in a way that the core is a polymer and the sheath carbon or vice versa, and with different cross-sectional shapes of the fibre core, their target properties are achieved. To achieve better electrostatic conductivity, the bicomponent CFs are woven in the warp and/or weft direction in the form of yarn composed of a fibre’s mixture or single strands in the form of multifilament, differently arranged in the fabric. Most often, their position in the fabric is in the weft direction due to simplifications in the fabric production process and to avoid possible deformations by stress and wear.

The results of analysis performed on the FE-SEM Mira LMU scanning electron microscope is shown in Figure 3. The CF cross-section clearly shows the core and the sheath and their differences formed during spinning. Carbon predominates in the darker part, while a certain polymer predominates in the lighter part. Thus, in group of samples 1–4, carbon predominates in the sheath, while in group of samples 5 and 6, it predominates in the fibre core.

CFs were spun in the form of filament (in samples 1–5) and in the form of cut fibre and afterword spun yarn (sample 6). Basic structural parameters of the woven fabric samples are shown in Table 1. It is clear that the fabric structural parameters of fineness and density of the warp and weft are the same for all tested samples, while the fabric mass and thickness, which are conditioned by the weave, differ minimally. During weaving (Table 2), CFs were woven as a multifilament in the weft direction (samples 1–3 and 5), in the warp and weft direction (sample 4) and as a (cut fibre) spun yarn in the warp and weft direction (sample 6). Based on the above, differences were obtained between the samples subjected to cyclic loads, on which the deformations of the samples in the warp and weft direction were investigated, as well as the sample electrostatic conductivity from fabric front to the fabric back side and vice versa.

### 3.2. Physical-Mechanical Woven Fabric Properties

The results of breaking forces and elongation at break before and after biaxial cyclic stress are shown in Table 3 and Table 4 and Figure 4 and Figure 5. Cyclic stresses affected the reduction of breaking forces in both stress directions (warp and weft direction) in all samples. The greater reduction in breaking forces is evident in the warp direction relative to the weft direction. If the direction of the warp (Figure 6) is observed, then sample 4 has the highest breaking forces and sample 1 the least. By observing the position of the CF it can be determined that it affect the breaking force. Sample 4 has CF as a multifilament yarn in the warp direction which affected the increase in breaking forces. A higher proportion of CF resulted in an increase in breaking force. After cyclic stresses, the order of the samples by breaking forces did not change, which means that sample 1 remained with the lowest breaking forces and sample 4 with the highest breaking forces. The results of the breaking forces in the weft direction also show that sample 4 has the highest breaking forces and an even greater difference compared to the other samples. The lowest values are in sample 3. Generally speaking, CFs increase the breaking force of fabrics before and after cyclic stresses. According to the error interval display, there is no significant deviation between the samples with a 95% confidence.

### 3.3. Woven Fabric Deformation

As the cyclic stresses increased, the deformations of the woven fabric samples increased (Table 5, Figure 6). Larger deformations after cyclic stress are present in the warp direction in almost all samples. Samples 1–3 show that by decreasing the proportion of CF in the weft direction (sample 1 has the largest proportion and sample 3 the smallest) the fabric deformations increase in the warp and weft direction. Sample 4 with CF in the warp and weft direction has the least deformation. The largest deformations were present mostly in samples 5 and 6 where the proportion of CF is the smallest.

### 3.4. Surface and Vertical Electrostatic Resistance

Surface and vertical electrostatic resistance of the samples measured from front to the back side of the fabric and vice versa are shown in Table 6 and Figure 7. The surface resistance in all samples is lower than the vertical. By measuring the electrostatic resistance from front to the back side and vice versa, no major differences are present, especially in the samples woven in twill 2/2 (samples 1–4). The differences between samples 1–4 are evident, which are manifested in the fact that a higher proportion of CF results in a lower electrostatic resistance. Sample 5, with a weft in which a CF thread is spun and woven in twill 1/3, shows a certain difference in surface electrostatic resistance between the front and the back side of the fabric (95.1 Ω/165 Ω), which indicates the impact of weave. Namely, the CF thread that is interwoven in sample 5 in twill 1/3 means that its presence is higher on the fabric front side, so it affects less resistance compared to the fabric back side where its presence is lower. Sample 6, which contains a cut CF twisted in the yarn used for warp and weft, and woven in twill 2/1, shows the similar surface resistance on the front and back side of the fabric (157.8 Ω/155 Ω). Since samples 5 and 6 are woven from the same CF group where carbon predominates in the core, their electrostatic resistance is higher than electrostatic resistance in samples 1–4, where carbon is predominant in the sheath.

Finally, it can be concluded that fabric samples containing a higher proportion of CF multifilament, with a higher carbon content on the fibre surface, as well as their position in the fabric, have a lower surface electrostatic resistance, i.e., better conductivity or better performance of protective fabrics against static electricity. This allows multiple applications of fabrics with a CF content that will satisfy not only the extremely important properties of static electricity conductivity but also the required strength and fabric durability.

### 3.5. Statistical Analysis

By correlation analysis of deformation properties and breaking force properties, the scattering of values by cyclic stress groups and the correlation coefficient in the warp direction (R = 0.6942) and in the weft direction (R^2^ = 0.6057) can be noticed, which confirms their interdependence, i.e., a relatively tight connection (Figure 8 and Figure 9). It can be concluded that a higher number of cyclic stresses affects a lower breaking force, i.e., a greater fabric deformation in the warp and weft direction. Greater scattering as well as lower interdependence of parameters is evident in the weft direction.

By correlating the parameters of electrostatic resistance and the proportion of CF in the fabric (Figure 10), it can be concluded that there is a relatively strong interdependence both in the case of surface resistance and in the case of vertical resistance, taking into account the front and back side of the fabric. As the proportion of CF in the fabric increased, the electrostatic resistance decreased, which is an extremely important indicator for the woven fabric design process for specific purpose of protective clothing.

## 4. Conclusions

In this research, six fabrics with different proportions and positions of bicomponent multifilament and cut carbon fibres (CF) were designed and woven, in order to observe the crucial parameters of woven fabric intended for protective clothing against static electricity. A relation between the proportion and position of CF in the fabric and the measured parameters (such as: physical and mechanical properties, fabric deformation before and after biaxial cyclic stress, surface and vertical electrostatic resistance from front to back side and vice versa) has been established. Using a new measuring device for biaxial cyclic stresses, new findings were made in defining the qualitative characteristics of fabrics with bicomponent CF. Fabrication of woven fabrics with different proportions of bicomponent multifilament CFs resulted in an almost linear relation of breaking forces and deformations of fabrics according to stress cycles.

By analysis of CF and considering their effectiveness on the removal of static electricity from the fabric it can be concluded that of significant effect is the following parameters: types of CF (composition and method of chemical spinning), their position in the fabric (warp, weft), the proportion of CF in the fabric, yarn structure (multifilament, spinning) and fabric weave.

Two types of bicomponent CF were selected with different proportions of carbon and other polymers in the fibre core and sheath and with different fibre cross-sections formed during chemical spinning. The position of the CF in the fabric samples was mostly in the weft direction. Greater effectiveness of protection against static electricity showed samples that had CF fibres woven into the warp and weft direction. The higher proportion of CF fibre in the fabric, especially in fabrics with multifilament, significantly affects the effectiveness of protection.

Applying a weave that has an equal representation of warp raisers and sinkers on the front and back side of the fabric (twill 2/2) shows a certain difference in the effectiveness of protection, where the front of the fabric has a slightly higher electrical conductivity i.e., lower electrostatic resistance. This points to the fact that the CF on the front side of the fabric create less resistance due to the flatter surface structure of the fabric. A higher number of warp raisers on the front of the fabric gives less surface electrostatic resistance.

According to the obtained results and statistical analysis, it can be generally concluded that fabric samples containing a higher proportion of multifilament CF, with a higher proportion of carbon on the fibre surface, as well as their position in the fabric (warp, weft) and weaves with a higher number of warp raisers on the front side, has lower surface electrostatic resistance, i.e., better conductivity or better efficiency of protective fabrics against static electricity.

This paper investigates newly developed woven fabric samples on the effectiveness of static electricity protection before and after biaxial cyclic stress on a patented device. This led to interesting results related to the effectiveness of protection against static electricity. The statistical comparison of individual parameters led to their mutual influences, which enable more accurate definition of the target properties of fabrics with a lower proportion of CF, which gives a certain scientific step forward in researching the effectiveness of protection against static electricity.

## Figures and Tables

**Table 1:** Basic structural parameters of woven fabric samples.

Sample	Tt (tex)		d (thread/cm)		Raw Material		m (g/m^2^)	T (mm)	Weave
	Warp	Weft	Warp	Weft	Warp	Weft			
1	20 × 2	20 × 2	38	21	50%PES/ 50%CO	50%PES/ 50%CO	228	0.67	Twill 2/2
2	20 × 2	20 × 2	38	21	50%PES/ 50%CO	50%PES/ 50%CO	244	0.62	Twill 2/2
3	20 × 2	20 × 2	38	21	50%PES/ 50%CO	50%PES/ 50%CO	258	0.56	Twill 2/2
4	20 × 2	20 × 2	38	21	50%PES/ 50%CO	50%PES/ 50%CO	240	0.69	Twill 2/2
5	20 × 2	20 × 2	38	21	50%PES/ 50%CO	50%PES/ 50%CO	278	0.57	Twill 2/2 * Twill 1/3 CF
6	20 × 2	20 × 2	38	21	60%MAC/ 39%CO/ 1%CF	60%MAC/ 39%CO/ 1%CF	256	0.58	Twill 2/1

Where: Tt (tex)—finesses, d (threads/cm)—density, m (g/m^2^)—mass, T (mm)—thickness; * twill 1/3 CF—weft threads with CF interlace with warp threads in weave twill 1/3.

**Table 2:** Position and proportion of CF in woven fabric.

Sample	Distribution and Proportion of CF in Fabric	Position of CF in the Fabric
1	Every 4th weft is a multifilament bicomponent CF. CF proportion: 12.5%	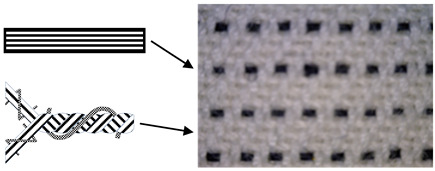
2	Every 12th weft is a multifilament bicomponent CF. CF proportion: 4%	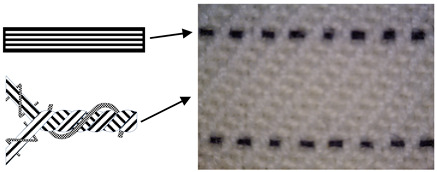
3	Every 24th weft is a multifilament bicomponent CF. CF proportion: 2%	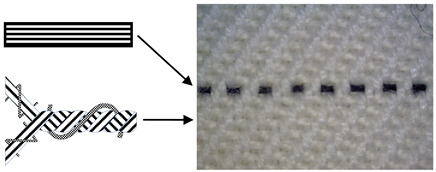
4	Warp: every 19th thread is a multifilament bicomponent CF. Weft: each 11th thread is multifilament bicomponent CF. CF proportion: 7%	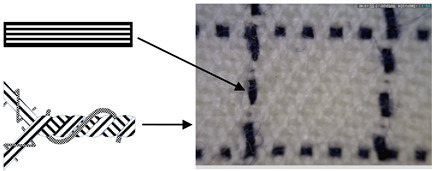
5	Every 12th weft is a 20 × 2 tex thread with one CF thread and one 50% CO/50% PES thread. CF proportion: 1%	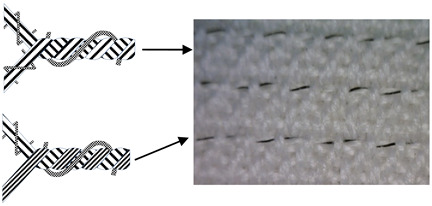
6	Mixed fibres in warp and weft, 60% MAC/39% CO/1% CF CF proportion: 1%	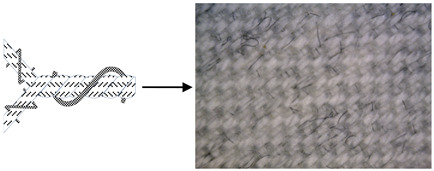

CF—carbon fibre, CO—cotton, PES—polyester, MAC—modacrylic fibre.

**Table 3:** Breaking force and elongation at break of samples in warp directions, before and after subjection to cyclic loads.

		Warp							
		Before Cyclic Stress				After Cyclic Stress			
Cycles	Samples	F (N)		Ɛ (%)		F (N)		Ɛ (%)	
		$\bar{X}$	CV (%)	$\bar{X}$	CV (%)	$\bar{X}$	CV (%)	$\bar{X}$	CV (%)
10,000 cycles	1	1646.7	9.4	20.7	7.5	1507.3	2.1	23.3	9.5
	2	1662.9	3.0	19.8	9.7	1521.1	6.3	20.1	9.1
	3	1675.2	2.1	21.1	7.6	1579.1	2.1	21.1	22.3
	4	1905.7	4.4	13.1	11.4	1886.0	5.3	13.7	11.1
	5	1774.5	8.5	22.4	9.5	1721.8	3.1	15.6	7.6
	6	1699.5	1.8	24.6	22.0	1580.2	4.7	15.4	9.7
20,000 cycles	1	1646.7	9.4	20.7	7.5	1386.3	3.8	15.5	25.2
	2	1662.9	3.0	19.8	9.7	1407.5	2.5	15.5	7.1
	3	1675.2	2.1	21.1	7.6	1487.4	3.9	20.5	6.8
	4	1905.7	4.4	13.1	11.4	1611.3	4.9	12.1	13.5
	5	1774.5	8.5	22.4	9.5	1626.9	4.6	15.9	6.4
	6	1699.5	1.8	24.6	22.0	1487.6	4.1	13.8	7.0
30,000 cycles	1	1646.7	9.4	20.7	7.5	1191.3	4.9	17.6	7.8
	2	1662.9	3.0	19.8	9.7	1258.6	4.9	19.4	11.0
	3	1685.2	2.1	21.1	7.6	1279.3	2.1	19.9	19.2
	4	1905.7	4.4	13.1	11.4	1444.7	3.6	11.5	10.3
	5	1774.5	8.5	22.4	9.5	1428.9	6.3	14.8	7.6
	6	1699.5	1.8	24.6	22.0	1299.6	2.1	13.7	20.9

$\bar{X}$—Mean value, CV—coefficient of variation (%).

**Table 4:** Breaking force and elongation at break of samples in weft directions, before and after subjection to cyclic loads.

		Weft							
		Before Cyclic Stress				After Cyclic Stress			
Cycles	Samples	F (N)		Ɛ (%)		F (N)		Ɛ (%)	
		$\bar{X}$	CV (%)	$\bar{X}$	CV (%)	$\bar{X}$	CV (%)	$\bar{X}$	CV (%)
10,000 cycles	1	873.3	3.1	18.2	2.3	793.3	4.1	17.6	8.5
	2	822.9	4.7	15.7	12.8	756.1	1.8	15.1	8.7
	3	758.7	3.8	19.1	8.5	789.4	2.1	18.8	10.6
	4	1145.7	4.7	14.7	7.4	941.9	3.5	15.6	11.8
	5	871.5	2.5	16.2	8.1	846.7	3.9	14.4	13.6
	6	906.7	3.9	18.7	11.6	866.7	4.5	18.2	11.6
20,000 cycles	1	873.3	3.1	18.2	2.3	729.2	9.4	16.6	13.3
	2	822.9	4.7	15.7	12.8	720.6	4.9	13.1	20.7
	3	758.7	3.8	19.1	8.5	678.6	4.1	17.5	9.9
	4	1145.7	4.7	14.7	7.4	916.2	2.7	11.3	11.5
	5	871.5	4.9	14.6	20.7	781.1	6.3	14.0	16.5
	6	906.7	7.3	16.6	9.9	820.3	4.1	16.2	15.8
30,000 cycles	1	873.3	5.6	17.2	16.5	684.8	5.3	12.6	11.4
	2	822.9	4.1	14.7	22.0	621.7	3.4	13.3	12.6
	3	758.7	6.4	15.6	15.5	596.1	4.6	14.6	9.1
	4	1145.7	4.7	14.7	7.4	714.7	4.4	8.7	10.8
	5	871.5	4.9	14.6	20.7	677.5	7.1	14.3	8.5
	6	906.7	7.3	16.6	9.9	701.1	5.5	13.2	14.9

$\bar{X}$—Mean value, CV—coefficient of variation (%).

**Table 5:** Woven fabric deformations in the direction of the warp and weft caused by cyclic stresses.

	Samples	Deformation (%)			
		Warp		Weft	
		$\bar{X}$	CV (%)	$\bar{X}$	CV (%)
10,000 cycles	1	1.82	1.20	1.14	0.76
	2	1.92	0.51	1.24	0.36
	3	1.99	1.14	1.88	0.58
	4	0.98	0.22	1.02	0.44
	5	2.11	0.19	2.09	0.33
	6	2.41	0.44	2.49	0.71
20,000 cycles	1	4.81	0.89	3.85	1.27
	2	4.68	1.20	3.71	1.19
	3	4.97	1.38	4.29	1.73
	4	3.97	1.26	3.49	1.55
	5	5.51	1.22	4.26	2.71
	6	5.01	1.47	4.14	0.87
30,000 cycles	1	9.45	1.59	8.32	2.07
	2	9.05	1.10	8.11	1.62
	3	9.61	1.56	8.85	1.77
	4	9.11	1.92	8.44	1.59
	5	10.41	2.52	9.01	1.84
	6	10.38	0.92	9.98	1.50

**Table 6:** Surface and vertical electrostatic resistance of the woven fabric measured from front to the back side.

Sample	Surface Resistance				Vertical Resistance			
	From the Front to the Back Side		From the Back Side to the Front		From the Front to the Back Side		From the Back Side to the Front	
	*ρ* (Ω)	CV (%)	*ρ* (Ω)	CV (%)	*ρ* (Ω)	CV (%)	*ρ* (Ω)	CV (%)
1	71.3	5.37	73.2	6.31	351.4	10.02	362.3	8.25
2	89.6	4.71	90.4	5.90	566.6	8.99	573.5	8.63
3	140.3	4.62	136.8	7.41	705.8	3.74	688.9	7.44
4	66.2	6.48	68.1	8.20	330.4	6.74	341.9	10.67
5	95.1	7.52	154.4	7.26	717.2	5.74	814.2	7.92
6	157.8	4.59	155.0	5.89	822.4	10.02	839.1	6.31
